# Insular dwarfism in horses from the Aegean Sea and the Japanese archipelago

**DOI:** 10.1007/s42991-024-00408-4

**Published:** 2024-03-29

**Authors:** Keesha M. Ming, Kévin Le Verger, Madeleine Geiger, Thomas Schmelzle, Georgios L. Georgalis, Genya Shimbo, Motoki Sasaki, Satoshi D. Ohdachi, Marcelo R. Sánchez-Villagra

**Affiliations:** 1 Department of Paleontology, University of Zurich, Karl-Schmid-Strasse 4, 8006 Zurich, Switzerland; 2 Naturmuseum St. Gallen, Rorschacher Strasse 263, 9016 St. Gallen, Switzerland; 3 Institute of Systematics and Evolution of Animals, Polish Academy of Sciences, Sławkowska 17, 31-016 Kraków, Poland; 4 Institute of Veterinary Medicine, Hokkaido University, Kita-19, Nishi-8, Kita-Ku, Sapporo, 060-0819 Japan; 5 Department of Veterinary Medicine, Obihiro University of Agriculture and Veterinary Medicine, Inada-Cho, Obihiro, Hokkaido 080-8555 Japan; 6 Institute of Low Temperature Science, Hokkaido University, Kita-19, Nishi-8, Kita-Ku, Sapporo, 060-0819 Japan

**Keywords:** Domestication, Allometry, Nanism, Bony labyrinth, Endocast

## Abstract

The horse (*Equus caballus*) varieties from Skyros and Rhodes islands (Greece) in the Aegean archipelago are extremely small, reaching shoulder heights of only about 1 m. Furthermore, the Japanese archipelago is home to eight small, native horse breeds. We investigated the evolutionary morphology and provided a review of historical documentations of these horses of cultural interest in Greece and Japan, thus providing a comparison of the independent evolution of small size in islands. We integrate cranial data from historical literature with data from newly gathered and curated skulls and analyse a measurement dataset featuring various domestic and mainland horse breeds and varieties. We use non-invasive imaging to study and measure 3D models of the bony labyrinth, housing the inner ear, and the braincase endocast. When considering the effects of allometry by regressing each PC1 scores (for each set of measurements) with the cranial geometric mean from linear measurements as a body size proxy, we show that size explains a large amount of the shape variation in horse crania, the bony labyrinths and brain endocasts. We found high intrabreed variation in all the analysed datasets. Globally, there are at least 30 distinct horse populations on islands, offering the chance to further study processes of convergence in morphological divergence and evaluate the effect of drift and the environment.

**Supplementary Information:**

The online version contains supplementary material available at 10.1007/s42991-024-00408-4.

## Introduction

Island dwarfism has attracted the attention of evolutionary biologists given their multiple convergent occurrences across the world and the possibility of disentangling processes of changes in body size (Van der Geer et al. 2021), associated with multiple ecological causes (Lomolino et al. 2012; Kolb et al. 2015). Some of the most iconic examples of island dwarfism concern proboscideans, hippopotami, and cervids, as recorded in Mediterranean Islands, and in the Japanese Archipelago in the case of deer and proboscideans (Van der Geer et al. 2021; Hayashi et al. 2023). These are mammals for which this phenomenon occurred naturally. There are other mammals that have repeatedly become insular artificially, such as horses (*Equus caballus* Linnæus 1758), ubiquitous across the world due to human activity. Horses have been transported to islands by humans, with at least 30 breeds or landraces of small-sized horses living on islands all over the world as recorded in our survey (Supplementary Materials 1, 2). Humans have thus facilitated repeated 'experiments' in size and morphological evolution associated with islands in a domesticated species (Masseti 2008; Hendricks 2007).

Body size in horses is highly variable, ranging from an average of less than one meter in the American Miniature breed to over two meters in Shire and Percheron breeds. Intensive selection for small size has created the American Miniature and other well-known miniature breeds such as the Falabella (Heck et al. 2019). Extreme size variation is a hallmark of the domestication process (Sánchez-Villagra 2022) and has been shown to affect horse cranial shape (Heck et al. 2018, 2019), likely affecting brain and inner ear gross morphology (Schutz et al. 2014; Schweizer et al. 2017; Costeur et al. 2019; Evin et al. 2022).

**Fig. 1:**
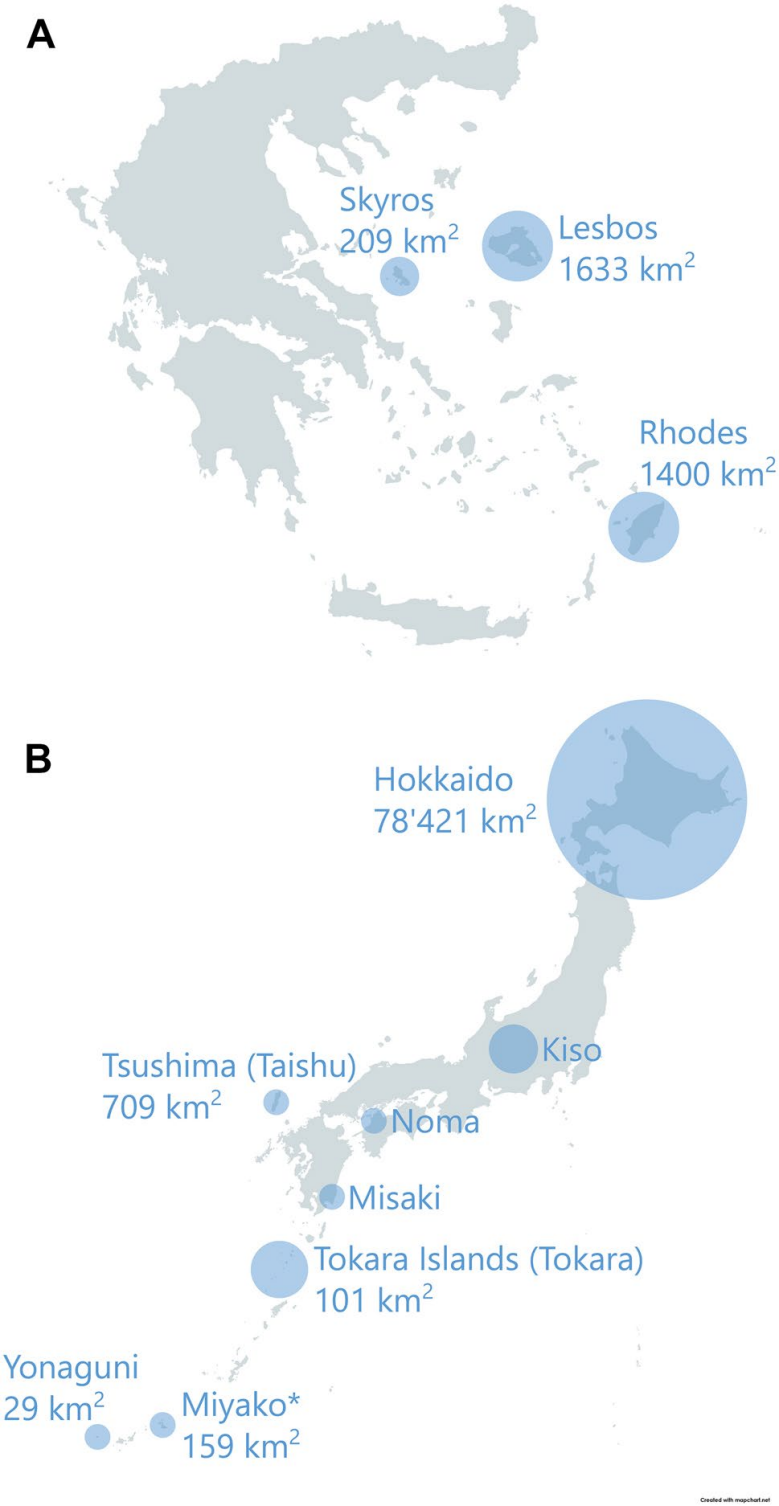
Home ranges of the studied Aegean breeds/varieties (**A**) and all native Japanese breeds (**B**). Insular home ranges include the size of the respective islands, excepted for the Japanese mainland. Maps adapted from mapchart.net. Symbol: *, The Miyako breed is the only native Japanese breed not represented by our study

The inner ear is located in the hollow bony labyrinth of the cranial petrosal bone, the space matching the shape and size of the membranous organs and soft tissues (e.g., Costeur et al. 2019). As an analogous internal imprint, the brain shape and size of mammals can be precisely studied reconstructing the virtual endocast, representing the space within the neurocranial cavity (Macrini 2006). These features and the external cranial shape can be analysed quantitatively with morphometric tools. As such, crania offer rich markers of morphological variation. Horse populations on islands from Greece and the Japanese archipelago (Fig. 1) offer the chance to study populations that have experienced great size reduction independently and repeatedly in each case.

### The small horses of Skyros, Rhodes, and Lesbos

**Fig. 2:**
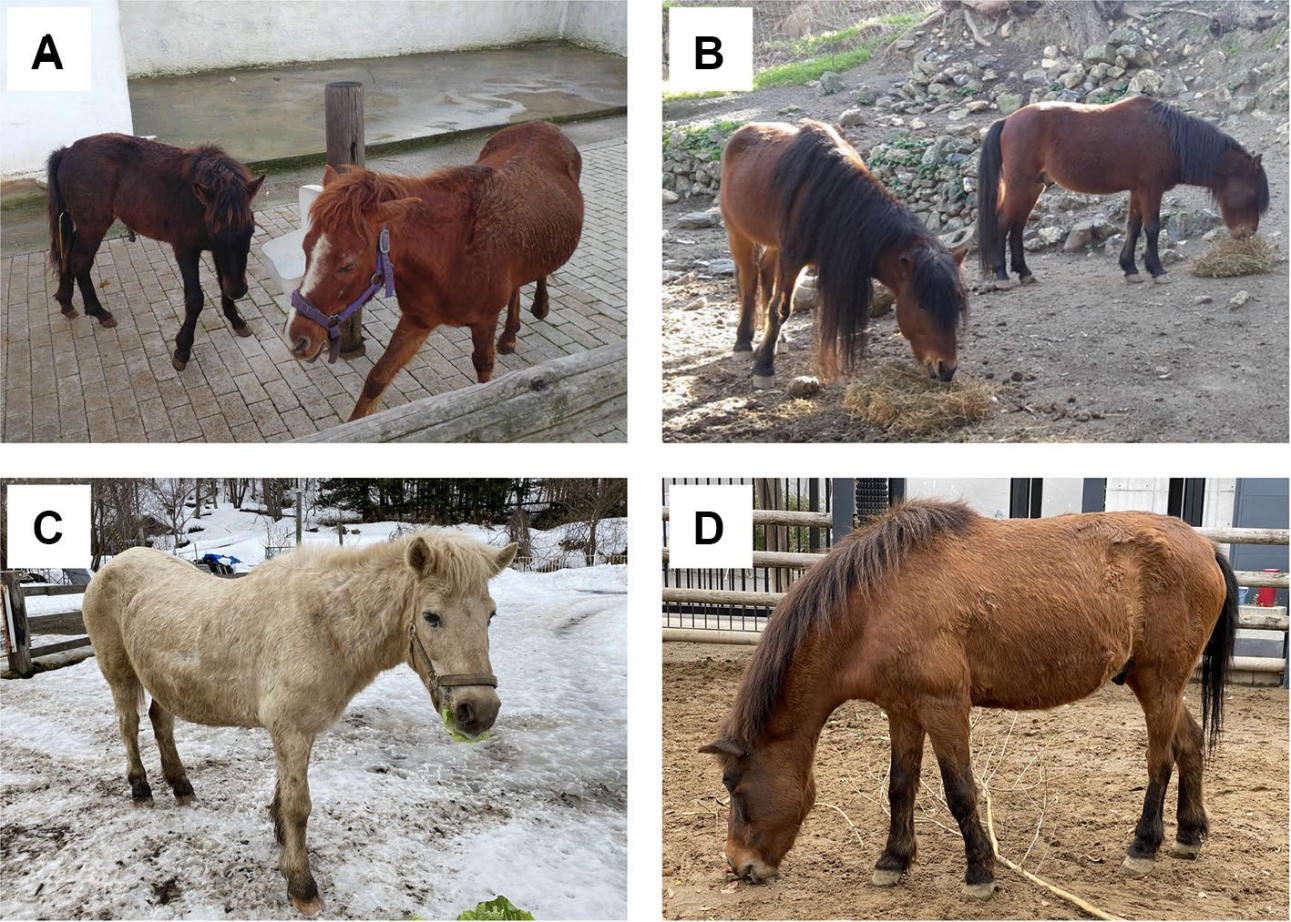
Examples of living Skyros horses (**A**), Rhodes horses (**B),** a Hokkaido horse (**C**), and a Tokara horse (**D**). Pictures taken by MRS-V, GLG, and KMM

The Skyrian and Rhodian small horses, as well as the horses from Lesbos Island, have been considered to be related and to belong to the so-called “Aegean horses”, which had a more widespread distribution in the past (Masseti 2012; Kostaras 2020). These horses stand out among the Aegean horse breeds due to their distinct phenotype (Zafrakas 1991; Kritikos 1994; Apostolidis et al. 2000, 2001; Hendricks 2007; Bömcke et al. 2011; Brown et al. 2013; Kostaras 2020), characterized by relatively small head and small ears (Kostaras 2020) (Fig. 2A). Specifically, the Skyros horse, of substantial recognition and popularity throughout Greece (Kostaras 2020), has even been considered as “the smallest ‘natural’ (feral) horse in the world” (Supplementary Material 3), being the product of natural isolation as opposed to selective breeding for small size like in the Falabella (Hendricks 2007; Kostaras 2020). The horses of Skyros were once abundant on the island; however, they have suffered a drastic reduction in their population through the loss of cultural traditions and potential competition with other grazing animals, leading the purebred individuals count to become only around 160 (Kostaras 2020; Supplementary Material 3). There are records of other breeds and varieties living in the Aegean region, notably those of Rhodes Island (Fig. 2B) and the “Midili” (or “Mytilinaiko” or "Mytiline") horse of Lesbos Island (or “Lesvos”), but, in comparison with the Skyrian horses or other breeds, these are inadequately known. As the Rhodes horse population is not officially registered as a breed at this point, we will continue referring to it as a variety, although it was treated as distinct in the recent compendium of the 'Network for the Protection of Greek Indigenous Farm Animals' (Amalthia 2020). The horse of Lesbos Island was until recently considered extinct, but a few living feral individuals still roam the island (Kostaras 2020). Even though most Greek citizens know of the Skyros horses, their remains are notoriously absent from museums, partially due to their protected status, but also due to their semi-wild living, meaning that individuals often die and decay in remote areas. The situation is even more dramatic for Rhodes horses (only 14 living specimens—November 2023) and horse of Lesbos Island, given the debate surrounding their status. One aim of this study is to emphasize the importance of curating specimens of rare breeds and varieties of domesticates, in an effort to preserve knowledge for future generations.

### The small horses of Japan

Japan counts eight small horse breeds related to different regions of the archipelago (average breed wither heights ranging from 102 to 142 cm): Hokkaido (Dosanko) (Fig. 2C), Kiso, Misaki, Miyako, Noma, Taishu (Tsushima), Tokara (Fig. 2D) and Yonaguni (Hayashida 1958; Hendricks 2007; Hartley Edwards 2016). The Japanese breeds have evolved to different conditions in their respective home ranges, and have typically been kept semi-wild, similarly to the Skyros and Rhodes horses (Kakoi et al. 2009; Tozaki et al. 2019). The Japanese breeds originated from Mongolian horses imported to Japan via the Korean Peninsula during the Kofun Period (third to fifth century AD) (Nozawa et al. 1998; Tozaki et al. 2003, 2019; Hendricks 2007), likely crossing Tsushima, and then spread across Honshu (mainland Japan), nowadays home range to only the Kiso breed. Some of the historical components regarding the modern breeds and their origin are known (e.g., Tozaki et al. 2019), but others are still unclear (Supplementary Material 3).

The Japanese breeds evolved for several centuries secluded from contacts outside Japan. In contrast, during the Meiji Era (1868–1912), there was a strong encouragement to integrate foreign breeds (e.g., the Thoroughbred and Arab, but also draft breeds like the Breton) into local horse populations in hopes of making them larger and sturdier, without success (e.g., Uzawa and Hongo 2006).

Currently, seven of the eight native Japanese breeds are endangered, with population sizes ranging between 40 and 138 individuals in 2017, the Hokkaido breed being the only breed with more than 1000 individuals (Tozaki et al. 2019). Similar to the Aegean horse breeds/varieties, museum specimens are rare to non-existent for most Japanese native breeds and the present study is a contribution to decipher their history.

We analyse the shape and size of the cranium, the bony labyrinth, and the brain endocasts to evaluate the impact of the island effect on morphological variation in populations of a domesticated species that has become independently feral in two archipelagos located on opposite sides of the Earth. Furthermore, by studying and comparing morphological data from horses of different islands with largely unrelated breed histories, we aim at finding convergent patterns of island dwarfing on cranial and endocranial structures.

## Materials and methods

We collected all the Aegean samples studied (MRS-V and GLG, several visits to the respective islands between 2018 and 2022), curated and deposited them at the Naturhistorisches Museum Bern, Switzerland. This includes 17 specimens of different ontogenetic stages, from 7 days to senile (Supplementary Material 4). We considered only skeletally and dentally mature (fully erupted third upper molars) individuals in the analyses: five Skyros horses, three Rhodes horses, and one Lesbos horse (Table 1). We studied data from a total of 19 Japanese adult specimens and one adult Korean specimen collected in several museums and institutions in Japan (Table 1). Of the eight official Japanese breeds, seven are represented by our dataset. One of these Japanese specimens (Yuigahama I, Table 1) is an archaeological sample from the fourteenth century (for a detailed description, see: Uzawa and Hongo 2006). We also studied an additional adult Japanese specimen, Yonaguni IV (TU_M33835). However, it was too incomplete to be included in the analyses. For the cranial analyses, we compared our specimens to the six Skyros horses from Dimitriadis (1937) and data for 138 individuals of 38 other domestic horse breeds and Przewalski’s horses, taken from Heck et al. (2018), leading to a dataset of 173 individuals. As one Skyrian individual measured by Dimitriadis (1937) (DIMI_4_H_sky_G) had incomplete data, this specimen was thus not included in the analyses. The final dataset for the cranial analyses corresponds to the full measurement data for 172 individuals (Supplementary Material 5).

**Table 1:** Demographic and collection information of the studied cranial specimens

Name	ID	Sex	Age	Locality	Housed at	EM	BLM
Skyros I	NMBE_1088728	Male	Mature	Skyros, Greece	NMBE CH	Yes	Yes
Skyros III	NMBE_1088730	Female	Mature	Skyros, Greece	NMBE CH	Yes	Yes
Skyros IV	NMBE_1088731	Male	15 years	Skyros, Greece	NMBE CH	Yes	Yes
Skyros VI	NMBE_1088739	Male	Mature	Skyros, Greece	NMBE CH	Yes	Yes
Skyros IX	NMBE_10996740	Male	Mature	Skyros, Greece	NMBE CH	Yes	Yes
Rhodes A	NMBE_1088733	Female	Senile	Rhodes, Greece	NMBE CH	Yes	Yes
Rhodes F	NMBE_1088738	Female	Mature	Rhodes, Greece	NMBE CH	Yes	Yes
Rhodes G	NMBE_1096739	Male	20 years	Rhodes, Greece	NMBE CH	Yes	Yes
Lesbos I	NMBE_1096741	Female	Mature	Lesbos, Greece	NMBE CH	Yes	Yes
Hokkaido I	23misc_1	Female	Mature	Kawahara's ranch, Memuro-cho, Hokkaido	OHD JP	Yes	Yes
Hokkaido II	OU_1	Female	Mature	NA (probably Hokkaido)	OU JP	Yes	Yes
Hokkaido III	BOT_13269	Female	Mature	NA (probably Hokkaido)	BOT HU JP	Yes	No
Tsushima I	OU_2	Female	Mature	NA	OU JP	Yes	Yes
Misaki I	KU_No.24	Female	8 years	Cape Toi, Miyazaki Pref	KU JP	Yes	No
Misaki II	KU_No.25	Male	14 years	Cape Toi, Miyazaki Pref	KU JP	Yes	No
Noma I	KU_Noma	Female	Mature	NA	KU JP	Yes	No
Tokara I	KU_T1	Female	Mature	Nakaoshima Island, Kagoshima Pref	KU JP	Yes	No
Tokara II	KU_T10	Male	Mature	Nakaoshima Island, Kagoshima Pref	KU JP	Yes	No
Yonaguni I	KU_No.2	Female	Mature	Kita-Bokujo ranch, Yonaguni Island, Okinawa Pref	KU JP	Yes	No
Yonaguni II	KU_No.3	Female	Mature	Kita-Bokujo ranch, Yonaguni Island, Okinawa Pref	KU JP	Yes	No
Kiso I	NU_00172	Female	33 years	Itsumiya Jinja Shrine, Minami-Kiso, Nagano Pref	NU JP	No	No
Yuigahama I	EM_Yui	Male	5–6 years	Fourteenth-century (Kamakura period), Yuigahama-minami Archeological Site, Kamakura City	EMJ	No	No
Kiso II	TU_M31212	Female	Mature	NA	TU JP	No	No
Yonaguni III	TU_M14385	Male	Mature	Shogo Inaba, Yonaguni Island, Okinawa Pref	TU JP	No	No
Hokkaido IV	TU_M77949	Male	Mature	Tama Zoo, Tokyo Pref	TU JP	No	No
Midget Horse I	TU_M65882	Female	28 years	Akiyoshidai Safari Land, Yamaguchi Pref	TU JP	No	No
Midget Horse II	TU_M33986	Male	Mature (Senile?)	NA	TU JP	No	No
Japanese Pony I	TU_M63162	Male	Mature (Senile?)	Saitama Childeren's Zoo, Saitama Pref	TU JP	No	No
Cheju I	EM_Cheju	Female	7 years	Cheju Island, South Korea	EMJ	No	No

*EM* endocast model, *BLM* bony labyrinth model, *NMBE CH* Natural History Museum Bern Switzerland, *OHD JP* Dr. Ohdachi Personal Collection Japan, *OU JP* Obihiro University of Agriculture and Veterinary Medicine Japan, *BOT HU JP* Botanical Garden of Hokkaido University Japan, *KU JP* Kagoshima University Japan, *NU JP* Nihon University Japan, *EMJ* Equine Museum of Japan, *TU JP* National Museum of Nature and Science Japan

We divided the domestic horses of the total cranial measurement sample into groups, to facilitate the analyses and comparisons of specific breeds and varieties. For the general results, we compared three groups: The Japanese horses (Hokkaido, Kiso, (Japanese) Midget, Misaki, Noma, Undefined pony (from Japan), Tokara, Tsushima, Yonaguni and the Yuigahama-minami horse), the Aegean horses (Lesbos, Rhodes, Skyros) and the comparative sample, made up of all other sampled breeds and feral horses.

In addition, we carried out the analyses of the cranial measurements with a focus on the visualization of specific breeds and varieties (Supplementary Materials 6, 7). These include each Japanese and Aegean breed/variety separately, but also other breeds and wild horses (Cheju pony, Exmoor pony, Falabella, Icelandic, Przewalski's horse, Shetland, and Scottish pony). All other breeds or feral horses were grouped into one large group, named “Horses”. The division is somewhat subjective but warranted due to our focus on specific populations that were previously mentioned to be “dwarfed” (Heck et al. 2019; Clauss et al. 2022), with specific domestication histories, and/or reported similarities among these breeds (Dimitriadis 1937; Hayashida 1958; Nozawa et al. 1998; Tozaki et al. 2003, 2019; Uzawa and Hongo 2006; Heck et al. 2019; Clauss et al. 2022).

### Bony labyrinth and brain shape as morphological markers

For the study of endocranial anatomy, i.e., here the brain endocast and the bony labyrinth, the sampling is concentrated only on the island horses focus of this study, including the nine Aegean horses mentioned above, as well as ten brain endocasts and three bony labyrinth models of Japanese horse crania (Table 1). The data to generate the 3D models of the Aegean brain endocasts and all of the herein studied bony labyrinths, for which the left petrosal bone of each cranium was removed for scanning (except Rhodes G (NMBE1096739) and Lesbos I (NMBE1096741), right petrosal), was acquired using an industrial micro focus CT scanner of the brand Nikon Metrology (Model XT H 225 ST) at the Irchel Campus of the University of Zurich. The Japanese brain endocast data were acquired by scanning the crania with an Aquilion Prime (Canon Medical Systems) located in the Faculty of Veterinary Medicine, Hokkaido University. Three-dimensional reconstructions were performed using Avizo v.2022.1 (Visualization Sciences Group, Burlington, MA, USA) by marking the area of every 10th slice and filling the gaps with Biomedisa (Lösel et al. 2020).

### Cranial, bony labyrinth and brain endocast morphometrics

**Fig. 3:**
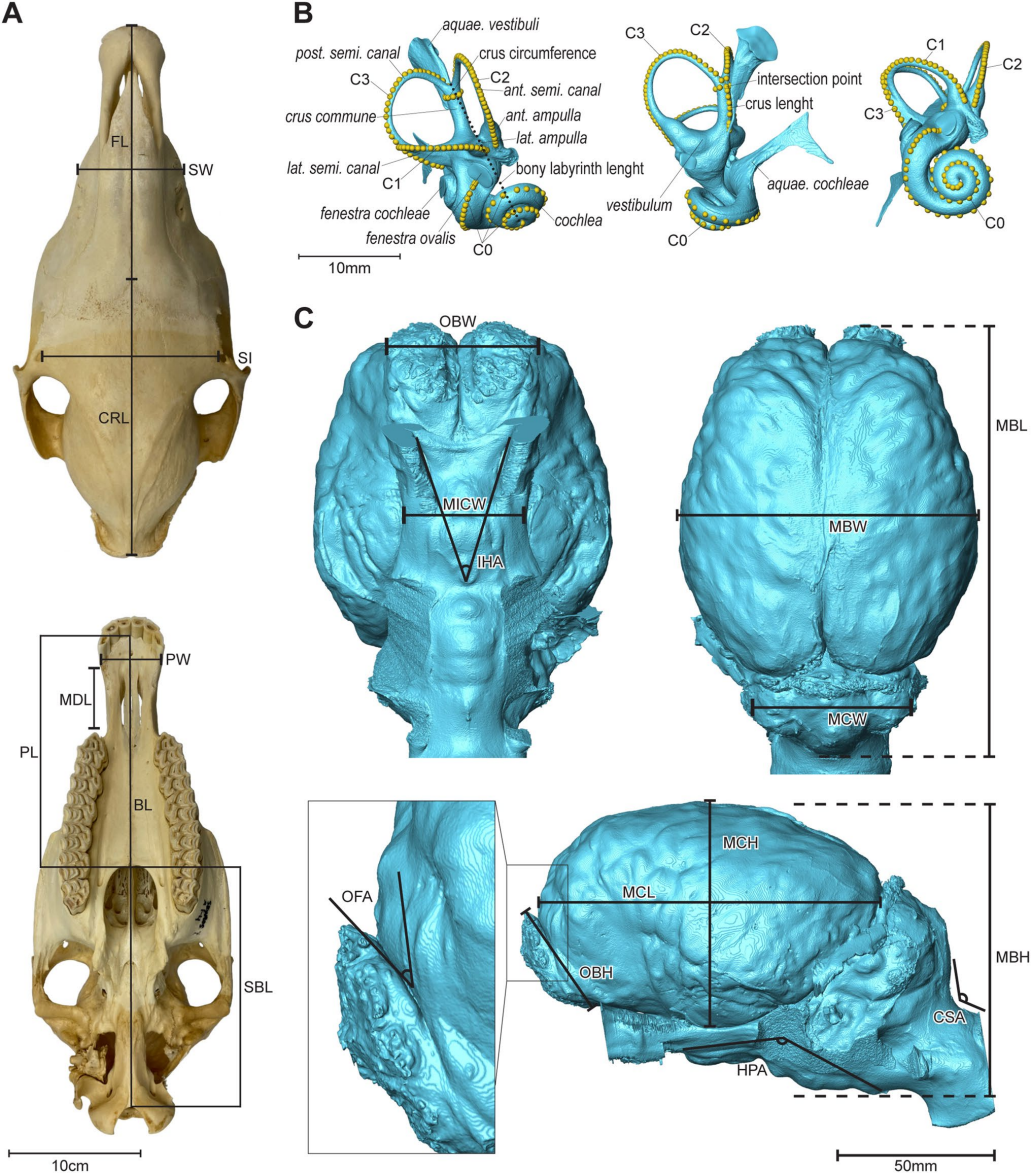
Measurements taken on the cranium (**A**), the bony labyrinth (**B**), and the braincase endocast (**C**). Cranial measurements illustrated on Skyros I (NMBE1088728) in ventral and dorsal views and modified from Heck et al. (2018). Measurements of the bony labyrinth of Rhodes A (NMBE1088733) in dorso-anterolateral, anterior, and anterolateral views. The landmarks here serve only to illustrate the measurements taken on the bony labyrinth. Brain endocast of Rhodes A (NMBE1088733) in ventral (**A**), dorsal (**B**), and lateral (**C**, **D**) views. As the right inner ear was removed for acquisition, missing parts appear on the right side of the braincase 3D mesh because of empty space during segmentation process. Measurements and their corresponding abbreviations are available in Table 2. Further abbreviations: *ant.* anterior, *aquae.* aquaeductus, *lat.* Lateral, *post.* posterior

Several linear measurements of the crania were taken, maximizing the number of comparable dimensions with both Dimitriadis (1937) and Heck et al. (2018). This was done through alignment of Dimitriadis’ (1937) descriptions of linear measurements with landmarks taken by Heck et al. (2018), and subsequent taking the interlandmark distance. However, some of the measurements had to be excluded due to missing data for incomplete crania, reducing the number of variables to nine measurements used in the subsequent analyses (Table 2, Fig. 3; Full data see Supplementary Material 5). For the definition of these variables, we followed the anatomical terminology of Von den Driesch (1976) when applicable. The measurements were taken with a digital caliper for measurements under 150 mm and a larger non-digital caliper for measurements over 150 mm. Because the measurement “Facial Length” was chosen to match the description of Dimitriadis (1937), rather than Heck et al. (2018), the measuring points in the Heck et al. (2018) dataset were approximated by the closest landmarks.

**Table 2:** List of measurements and their anatomical descriptions

Abbreviation	Measurement name	Corresponding measurements in literature	Anatomical description
*Cranium*			
BL	Basilar length	Basilarlänge (D), 60–37 (H)	Distance between the most anterior point of the foramen magnum (Basion) and the most anterior point of the palate between the incisors on the midline in ventral view
SBL	Skull base length	For. Magnum—Choanenrand (D), 60–46 (H)	Distance between the most anterior point of the foramen magnum (Basion) and the most posterior point of the palate on the midline in ventral view
PL	Palatal length	Choanenrand—mittl. Inz. (D), 46–37 (H)	Distance between the most posterior point of the palate and the most anterior point of the palate between the incisors on the midline in ventral view
MDL	Maxillary diastemal length	Diastemalänge (D), 42–2 (H)	Distance between the most mesial point of the left toothrow at the alveolar base (P2) and the most labio-distal point of the upper third incisor (I3) at the level of the alveolar base in ventral view
PW	Premaxillary width	Schnauzenbreite (hinter den Schneidezähnen) (D), 2–1 (H)	Distance between the most labio-distal points of the left and the right third Incisors (I3) at the level of the alveolar base in ventral view
SI	Supraorbital interdistance	Breite zwischen der for. infraorbitalia (aussen) (D), 7–8 (H)	Distance between the most medial point of the left and the right supraorbital foramen in dorsal view
CRL	Cranial roof length	Kraniallänge nach Salensky (D), 33–11 (H)	Distance between the occipital protuberance and the nasal–frontal suture (Nasion) on the midline in dorsal view
FL	Facial length	Faziallänge nach Salensky (D), 11–37^a^ (H)	Distance between the nasal–frontal suture (Nasion) and the most anterior point of the premaxilla on the midline (Prosthion) in dorsal view
SW	Snout width	Breite zwischen dem vorderen Ende der crista maxil. (D), 9–10 (H)	Distance between the most anterior point of each facial crest in dorsal view
*Bony labyrinth*			
C0	Length of the cochlea	C0^a^ (CL)	From the cochlear apex to the base of the oval window
C1	Length of the lateral semicircular canal	C1 (CL)	From the junction between lateral and posterior semicircular canals to the base of the lateral ampulla
C2	Length of the anterior semicircular canal	C2 (CL)	From the base of anterior ampulla to the IP
C3	Length of the posterior semiscircular canal	C3 (CL)	From the IP to the base of the posterior ampulla
Bony labyrinth length	Bony labyrinth length	Bony labyrinth length (CO)	Proxy for overall size of the inner ear: From the cochlear apex to the top of the *crus commune*
*Crus* length	Length of the *crus commune*		From the IP to the attachment site of the cranial nerve VII
*Crus* circumference	Circumference of the *crus commune*		Circumference of the *crus commune*: IP as starting- and endpoint
*Brain endocast*			
MBW	Maximal braincase width	Brain width (DA)	Distance between the most lateral points on each side of the cerebrum in dorsal view
MBL	Maximal braincase length	Brain length (DA)	Distance between the most posterior point of the cerebellum and the most anterior point of the olfactory bulbs in dorsal view
MCL	Maximal cerebrum length	Cerebrum length^a^ (DA)	Distance between the most posterior and anterior points of the cerebrum in lateral view
MBH	Maximal braincase height	Brain height^a^ (DA)	Distance between the most ventral point of the medulla oblongata and the most dorsal point of the cerebrum in lateral view
MCH	Maximal cerebrum height	Cerebrum height (DA)	Distance between the most ventral and dorsal points of the cerebrum in lateral view
OBW	Olfactory bulbs width		Distance between the most lateral points on each side of the contact between olfactory bulbs and cerebrum in ventral view
MICW	Minimal intercanal complex width		Distance between the points of the maximal concave curvature on each lateral border of the intercanal complex (oval canal—optic canal—sphenorbital fissure) in ventral view
MCW	Maximal cerebellum width		Distance between the most lateral points on each side of the cerebellum in occipital view
OBH	Olfactory bulbs height	Olfactory bulb length (DA)	Distance between the most ventral and dorsal points of the contact between the olfactory bulbs and the cerebrum
IHA	Intersphenorbital–Hypophyseal Angle		Angle defined by the deviation of the medial edge of the sphenorbital fissures from the notch of the hypophyseal region in ventral view
HPA	Hypophyseal–Pons Angle	Angle medulla oblongata and orbitary nerves (II) (DA)	Angle defined by the posteroventral orientation of the pons with respect to the ventral plane of the hypophyseal region in lateral view
CSA	Cerebellum–spinal cord angle	Angle cerebellum and medulla oblongata (DA)	Angle defined by the posterior plane of the cerebellum with respect to the plane formed by the spinal cord in lateral view
OFA	Olfactory bulbs–frontal lobe angle	Angle frontal lobes and olfactory bulbs (DA)	Angle defined by the plane formed by the posterior plane of the olfactory bulbs with respect to the anterior plane of the frontal lobe in lateral view

Most of the selected cranial measurements recover both the distances taken by Dimitriadis (1937) and the interlandmark distances of Heck et al. (2018). Only the "Facial Length" preferentially follows Dimitriadis (1937). The bony labyrinth measurements follow Clavel et al. (2021) and Costeur et al. (2019), and brain endocast measurements Danilo et al. (2015), unless indicated with an asterisk. Crus length, Crus circumference, Olfactory Bulbs Width, Minimal Intercanal Complex Width, Maximal Cerebellum Width, and the Intersphenorbital–Hypophyseal Angle were newly defined. All bony labyrinth measurements were taken on the surface of the models

*D* Dimitriadis (1937), *H* Heck et al. (2018), *CL* Clavel et al. (2021), *CO* Costeur et al. (2019), *DA* Danilo et al. (2015), *IP* Intersection point of the anterior and posterior semicircular canals

^a^Modified from the original measurement

For the linear measurements on the bony labyrinth (Table 2, Fig. 3), we predominantly followed the approach of Clavel et al. (2021), adding three more measurements, two newly defined and one analogous to one variable in Costeur et al. (2019). We measured the length of the cochlea (C0) in a different way than Clavel et al. (2021) by starting at the apex and following the line up to the base of the *fenestra ovale*. C1–C3 were measured analogously to Clavel et al. (2021). The two newly defined measurements concern the circumference of the *crus commune* at the intersection point, and its length from the intersection point to the base of the *aquaeductus vestibuli*. The one analogous to Costeur et al. (2019) is the bony labyrinth length, measured from the apex of the cochlea to the highest point at the intersection of the posterior and anterior semicircular canals.

Following the work on equid brain endocasts of Danilo et al. (2015), we took some of the linear measurements and angles they defined, and added four new ones (Table 2, Fig. 3). Adjustments from Danilo et al. (2015) were made to the measurements of cerebrum length, excluding the olfactory bulbs, and the braincase height, measuring up to the *medulla oblongata*. In total, we end up with 13 measurements for the 19 brain endocast models.

### Multivariate analyses

The markers of morphological variation in the investigated domestic horses were shape variation of the cranium, the brain endocast, and the bony labyrinth, where shape is defined as all the geometric features of an object except its size, position and orientation (Dryden and Mardia 1998). We analysed (a) with raw data (i.e., the form of each structure defined by the combination of shape and size); (b) with standardized data, called shape ratios, to remove size effect (see Claude 2008); and (c) with data corrected for the allometric effect (also called allometry-free shape). For part (b), because we have no data on body size for the studied specimens and we shouldn't arbitrarily choose one variable as a proxy for size, the geometric mean (GM) (Mosimann 1970; Claude 2008), i.e., the nth root of the product of n measurements, was used as a general proxy for body size throughout our analyses, calculated with all nine cranial measurements. Thus, the log shape ratios correspond to the log of the ratio of each measurement with the GM of each related specimen (Claude 2008). We included three angle measurements in the brain endocast dataset and those were not standardized. For part (c), we removed shape variation due to allometry by extracting the residuals from a MANOVA of the set of raw variables of a given object against the GM, with the base R *manova* function.

Form, shape, and allometry-free shape variations for each anatomical structure (i.e., cranium, brain endocast and bony labyrinth) were explored with Principal Component Analyses (PCA), using the *prcomp* function, to assess the distribution of specimens in the morphospace along the two main PC axes for the three above-mentioned parts (i.e., a, b, and c). As shape variation is assumed to be highly correlated with size variation due to the insular dwarfism, we evaluated the allometry for the cranium, brain endocast and bony labyrinth, separately. Due to the number of measurements for each dataset, we didn't analyse allometry conventionally in a myriad of bivariate analyses but used a multivariate approach to consider all variables simultaneously, based on Jolicoeur (1963) (see Klingenberg (1996) for a list of examples, and Klingenberg (2016) for a methodological review concerning allometry). To do so, as the PC1 axis exhibits the highest amount of total variance and present a size gradient in morphospace (see “Results” section), we extracted the scores of the PC1 axis from the analysis with the log shape ratios (= part b) for each anatomical structure to perform a linear regression with the log GM (Claude 2008; Klingenberg 2016). Subsequently, using the *lm* function of the package *stats*, we evaluated whether the variation in size statistically affects the shape of each anatomical structure (*p* value < 0.05) with an ANOVA and, if so, we extracted the correlation coefficient (*R*^2^) between shape and size, following a linear model. Thus, allometric variation is assessed on a large sample of horses for the cranium and restricted to the new horses from the present study (Table 1) due to a lack of available data for the endocranial anatomy of other breeds. The PCA loadings were added to each plot to highlight the impact of the variables on each axis and facilitate interpretation. All analyses were conducted using R (Version 4.3.0; R Core Team 2023).

## Results

### Cranial shape analyses

**Fig. 4:**
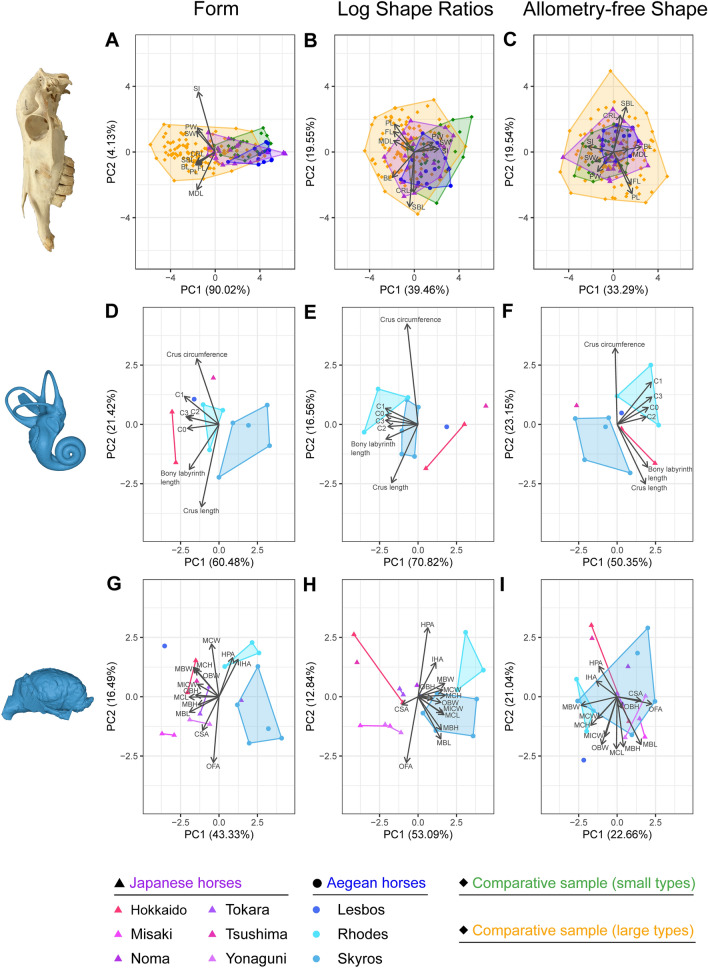
Principal component analyses and loadings of cranial, bony labyrinth and endocranial shape in the investigated horses (PC1-2). Cranial morphospace with raw (= form) (**A**), log shape ratios (**B**) and allometry-free shape (**C**) data. Bony labyrinth morphospace with raw (= form) (**D**), log shape ratios (**E**) and allometry-free shape (**F**) data. Braincase endocast morphospace with raw (= form) (**G**), log shape ratios (**H**) and allometry-free shape data (**I**). Abbreviations for each variable are available in Table 2

On the PCA for form changes, the PC1 accounts for 90.02% of the variation and PC2 for 4.13%. The Supraorbital Interdistance and the Maxillary Diastemal Length (Table 2) have the strongest effect on the positive and negative PC2 values, respectively. All measurement loadings extend towards the negative PC1 values, indicating that an increase in any measurement leads to more negative PC1 scores. This strongly suggests that the PC1 axis reflects overall size variation. The morphospace of the first two principal components (PCs) (Fig. 4A) shows a positioning of the Aegean and Japanese horses on the positive axis of PC1, but largely overlapping with the comparative sample. When comparing the individual breeds and varieties among each other (see Supplementary Material 6), the morphospace of the first two PCs shows a gathering of the Skyros and Rhodes horses with the two previously defined “dwarfed” breeds of Shetlands and Falabella, as well as the Japanese Midget horses and Undefined Pony on the positive values of the PC1 axis. The other Japanese horses (Hokkaido, Kiso, Misaki, Noma, Tokara, Tsushima, Yonaguni and the Yuigahama-minami horse) and the Cheju pony take a position closer to the Scottish ponies and the Icelandic and Przewalski’s horses but are still overall grouped on the positive values of PC1.

The first two principal components on log shape ratios account for 39.46% and 19.55% of the shape variation (Fig. 4B). Having standardized all measurements, their loadings' orientation in the morphospace of the first two PCs have changed as well. Skull Base Length and Cranial Roof Length (Table 2) exert the strongest influence overall, both extending towards the negative PC2 scores. The three groups overlap much more in the morphospace than with the form data, but the Aegean group is generally still situated on the positive values of PC1, mostly explained by Premaxillary Width, Supraoccipital Interdistance, and Snout Width (Fig. 4B). Together, these three variables indicate that the positive PC1 values correspond to a relatively wider face.

From allometry-free shape morphospace, PC1 accounts for 33.29% and PC2 for 19.54% of the variation (Fig. 4C). While Skull Base Length and Cranial Roof Length still exert a strong influence on the PC2 coordinates, Palatal Length and Facial Length gained strength as well through the removal of allometry, extending towards the opposite direction of Skull Base Length and Cranial Roof Length (Table 2). The three groups strongly overlap and are situated around the center of the first two principal component axes (Fig. 4C). On the PC1, specimens are distributed according to a relatively wider face on the negative PC1 values (i.e., Premaxillary Width, Supraoccipital Interdistance, and Snout Width) and a relatively longer basilar length (BL) and maxillary diastema (MDL) on the positive PC1 values.

In the PCA showing the different breeds and varieties (Supplementary Material 6), all groups are more centered within the “Horse” group than in the form and the log shape ratios PCAs, except for one Hokkaido specimen (Hokkaido IV, TU_M77949) just outside the border of the “Horse” group. Notably, the Japanese breeds overall vary greatly along PC2 within their groups, except for the Hokkaido breed, that shows a larger diversification along PC1 than PC2, and the solitary Noma specimen on the positive side of PC1, that does not overlap with any other highlighted group but is closest to the Rhodes group.

Combined, these three morphospaces show that the morphological distance between the crania of the dwarf horses of the Aegean and Japanese islands to the comparative sample seems to be mainly explained by allometry.

**Fig. 5:**
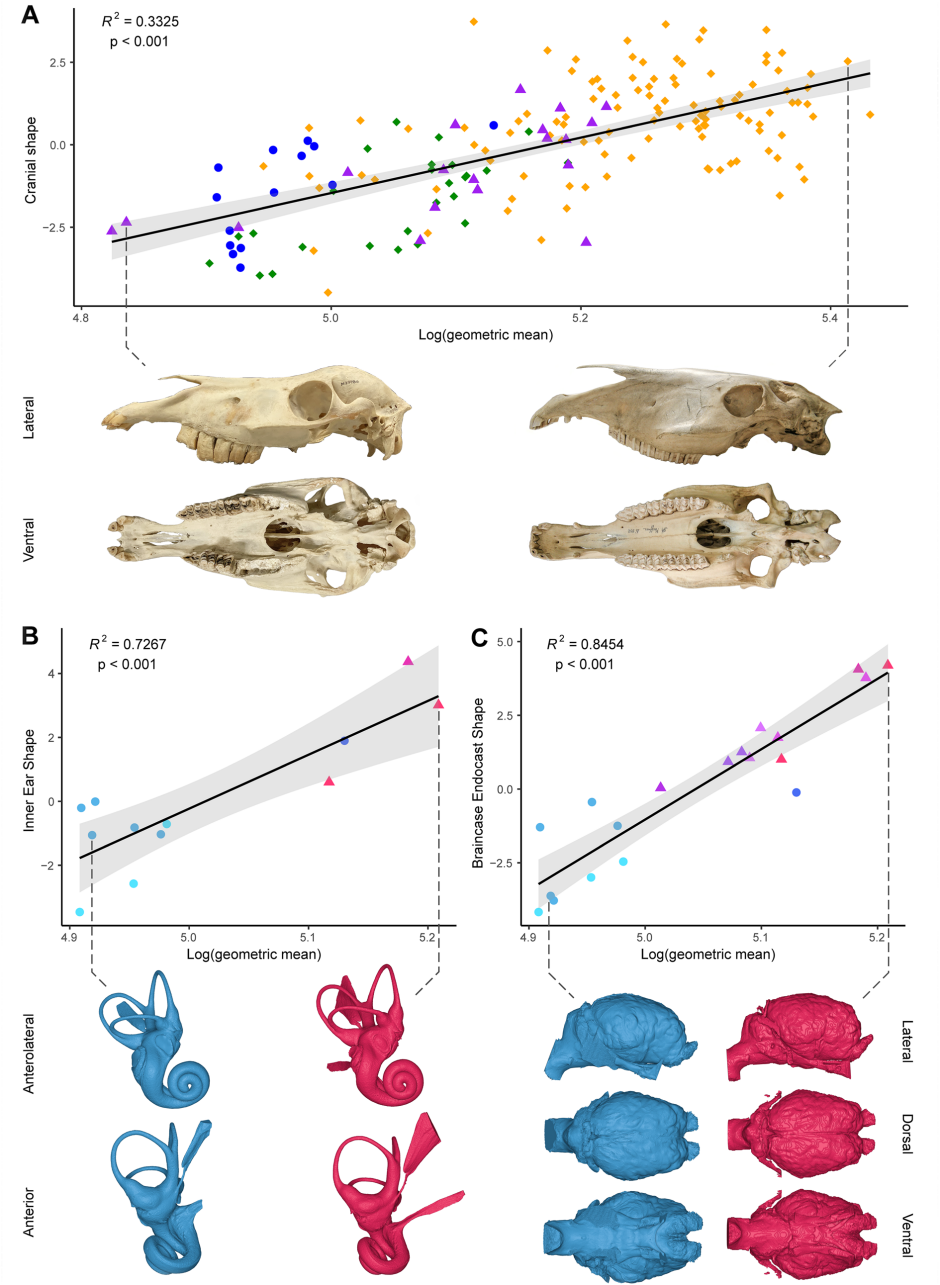
Multivariate regressions of the PC1 scores from the log shape ratios PCA with log(geometric mean) for the cranium (**A**), the bony labyrinth (**B**), and the brain endocast (**C**). Gray spectrum indicates a 95% confidence interval. Illustrated individuals are TU_M33986 and V_000E931_H_piz_C_3_6 for (**A**) and NMBE_1088739 and OU_1 for (**B**, **C**). Color legend refers to Fig. 4

Cranial shape is strongly influenced by size variation, explaining 33.25% of the morphological variation extracted from PC1 scores on the log shape ratios (Fig. 5A). Small horses (i.e., the Aegean and Japanese horses, as well as some selected breeds in the Supplementary Material 7) and the comparative sample follow the regression slope relatively consistently. While larger horses show a lengthening of the rostrum, smaller horses exhibit a relative transverse widening of the rostrum in comparison. The morphological variation associated with size variation in horses is thus mainly carried by the muzzle (Fig. 5A), which is also reflected by the loadings shown in Fig. 4C reported above.

### The shape of the bony labyrinth

The PCA for the investigation of form changes is composed of a PC1 accounting for 60.48% and a PC2 explaining 21.42% of the total variance (Fig. 4D). With log shape ratios (Fig. 4E), PC1 and PC2 account for 70.82% and 16.56% of the total variance, respectively. Finally, Fig. 4F shows the allometry-free shape analysis with a PC1 accounting for 50.35% and a PC2 explaining 23.15% of the total variance.

Although the small sample size calls for caution, in all morphospaces, the five breeds never overlap, suggesting a potentially unique shape for each group in our sample, supposedly less dependent on size variation. However, we observe a relocation of the groups in the comparison of each morphospace, more specifically for the Hokkaido, Tsushima and Lesbos specimens. In particular, the Japanese dwarf horses—slightly larger than the Aegean dwarf horses—focus on the positive values of PC1 of the log shape ratios, whereas the latter occupy an opposite position on negative PC1 values mostly supported by relatively longer semicircular canals and cochlea (Fig. 4E).

However, bony labyrinth shape extracted from the PC1 scores of the log shape ratios is highly correlated with size (72.67% of the shape variation, Fig. 5B). All small horses considered together define a regression slope, but it could be driven by the separation in two groups, maybe because of the low sample size: the first, the smallest, is made up of Skyrians horses and Rhodes horses, and the second, the largest, is composed of Lesbos horse and Japanese specimens. The major allometric variation between the two groups corresponds to a relative increase in the length of the semicircular canals and the cochlea, as well as an increase in the length of the inner ear, in the Skyrians and Rhodian horses compared to the Lesbos horse and Japanese specimens (Fig. 5B).

### The shape of the braincase endocasts

The PCA on form show PC1 and PC2 explaining 43.33% and 15.43% of the total variance, respectively (Fig. 4G). Most groups do not overlap, and, as for the bony labyrinth, Japanese horses have a morphospace occupancy in the negative values of PC1, except for the Noma specimen, while Aegean horses exhibit an opposite morphospace occupancy on PC1, except for the Lesbos horse. This difference in morphospace occupancy is even more marked for the PCA on the log shape ratios (Fig. 4H), for which PC1 and PC2 account for 53.09% and 12.84% of the total variance, respectively. Standardizing braincase endocast data therefore tends to discriminate between the Aegean Sea and the Japanese Archipelago horses on PC1, Skyros and Rhodes horses distributing on positive PC1 values due to the relatively smaller dimensions of the braincase as a whole, with the exception of a relatively larger cerebellum (MCW) and Intersphenorbital–Hypophyseal Angle (IHA).

In the morphospace of the allometry-free shape PCA (Fig. 4I), for which PC1 accounts for 22.66% and PC2 for 21.04% of the total variation, the distinction detected after standardization between the Aegean and Japanese specimens is not observed, underlining that the segregation we observe in Fig. 4H is largely due to allometry. The comparison of all morphospace is in this regard similar to what is detected for the cranium.

Indeed, the regression of the PC1 scores from the log shape ratios on the log(GM) (Fig. 5C) shows that brain shape is strongly influenced by size variation, explaining 84.54% of the shape variation on PC1. As for the bony labyrinth, a distinction of Aegean and Japanese distribution along the slope is detected. While Japanese horses are more clearly characterized by a relative increase in the angle between the cerebellum and the spinal cord, and the angle between the olfactory bulbs and the frontal lobe, Aegean horses show an increase in all other variables, highlighting a relatively larger braincase as cranial size decreases in our sub dataset (Fig. 5C).

## Discussion

We have analysed the form, shape, and allometry-free shape of the cranium, the bony labyrinth, and the brain endocasts and assessed differences and similarities between several horse breeds/varieties and landraces in the context of island-living of domesticated animals. The differentiation of island small horse breeds may be associated with the breed history, and morphologically reflect differences from small size resulting from selective breeding. The Aegean breeds/varieties and landraces studied here, as part of an Aegean historical and geographical cluster, have been living semi-wild on their corresponding islands for at least a few centuries, as have the diverse Japanese horses studied. In contrast, the Falabella breed is a relatively young one, dating back to the mid nineteenth century, strictly managed and cross-bred with small horse breeds (including the Shetland breed) for several generations and selectively bred for small size (Hendricks 2007).

### The shape of the cranium

Some previous ideas on the Aegean horses that we studied and compared with other horse breeds are confirmed in our study. Dimitriadis (1937) noted similarities among the Skyrians, the Shetlands and the Icelandic, which overlap in our results (Supplementary Material 6). Furthermore, Clauss et al. (2022), who studied the skulls of some of our Skyrian and Rhodes specimens, also noted similarities with the Shetlands and the Falabella regarding the size and shape of the cranium and the mandible, also in relation to body size. This is unsurprising, as the Shetland breed is included in the Falabella breed ancestry (Hendricks 2007). Our results indicate this as well, with the Falabella falling into the shape-space of the Shetlands. However, when allometry is removed, this specimen also falls into the shape-space of the Icelandic breed, the Hokkaido breed and the Przewalski’s horses, thus, its similarities to the Shetland are not exclusive. The Aegean horses also show similarities to some of the Japanese breeds, as the Aegean shape-space falls within the Japanese shape-space when allometry is removed, and some individuals are situated very closely to each other within the morphospace. However, as for the noted similarities of the Skyrians with some other highlighted breeds and varieties, the similarities between the Japanese and Aegean horse crania are not exclusive, and many specimens of the comparative sample share their shape-space as well. Nevertheless, an important result can be highlighted, namely that although the Aegean and Japanese horse breeds have different histories, the morphological differences are subtle, with both groups appearing to be subject to cranial allometry (Fig. 5A). Such a result suggests a strong pattern of morphological convergence among the dwarf horses of the islands, whose differences are the result of their size variation. This hypothesis could be explored by comparing allometric trajectories between different horse breeds with better sampling.

When each breed is investigated in detail (Supplementary Material 6), our study coincidentally depicts some previously reported groups of native Japanese horse breeds. Hayashida (1958) clustered “Small-sized” versus “Medium-sized” horse breeds according to body size, and Tozaki et al. (2019), found two genetic clusters based on 9700 SNP loci: The Japanese mainland lineage (Hokkaido, Kiso, Misaki, Tsushima and Noma) and the southern islands lineage (Tokara, Miyako and Yonaguni). Although limited by the sample size, implying caution in interpretation, when log shape ratio and allometry-free shape analyses are considered, our results show Misaki and Noma are situated closer to the southern islands lineage individuals (i.e., Tokara and Yonaguni), rather than with the mainland lineage individuals (*contra* Tozaki et al. 2019). Furthermore, the Kiso breed occupies an intermediate position between our groups, suggesting morphological similarities with both lineages, in fact, a geographically intermediately located group. As the Cheju horses are also reported to have originated from the Mongolian horse lineage, like the Japanese breeds (Nozawa 1998), their grouping in the morphospace with some of the earliest immigrating horse breeds (i.e., the mainland lineage) was the null hypothesis. Because we have a low sample size for most Japanese breeds, we cannot rule out the possibility that this grouping is pure chance. Nevertheless, future studies including more specimens could verify if morphological variation indeed can be visualized through geographic distribution, mirroring patterns usually found through genetic studies.

Despite the difficulties of reporting patterns for low sample sizes, some results are worth noting. The archaeological specimen from the Yuigahama-minami tomb (fourteenth century, Kamakura Period), with a withers height that is estimated to be at 140 cm, is the largest among the known medieval horses of Japan. Its cranial morphology was previously reported to be analogous to that of the modern native Japanese breeds, except the Kiso breed (Uzawa and Hongo 2006). However, taking size effect and allometry into account, we found that the Yuigahama-minami specimen falls into the shape space of the Hokkaido breed and shows a similar cranial morphology to the Japanese Midget horses, the Japanese Undefined pony, and the singular Tsushima and Cheju individuals (Supplementary Material 6). The Yuigahama-minami specimen is even situated closer to some of the Kiso specimens than to other Japanese specimens, such as the Misaki, Noma, Tokara and Yonaguni specimens. Therefore, the Yuigahama-minami specimen may have a more similar cranial shape to at least some specimens of the Kiso breed than previously reported by Uzawa and Hongo (2006).

This result also portrays another characteristic phenomenon of the native Japanese horses: their seclusion until the Meiji restoration. The Kiso breed specifically was previously reported to have been morphologically influenced by incorporation of foreign horses into the breeding programs during the Meiji restoration, becoming larger and adopting traits of other breeds (Takasu et al. 2011). However, it has been shown that since 1948, the Kiso breed has become smaller again through backcrossing, suggesting that the breed may experience a reversal to a similar morphology like before the Meiji era (Takasu et al. 2011). We do not know if the body size of this breed has further changed in more recent times, because we do not have the exact death dates for both post-Meiji Kiso specimens included in our study. Nevertheless, this breed has probably undergone unique combinations of morphological changes in a short amount of time. Alongside including more spatially distributed specimens, future studies may incorporate more temporally distributed specimens, such as pre-Meiji Kiso specimens, to better understand morphological changes across time.

### The shape of the bony labyrinth

The effect of size on the shape of the bony labyrinth leads to contrasting results. If two groups are considered, such as the Greek and Japanese horses, the allometry is supported by the position of the Lesbos horse according to other specimens. However, along the regression slope, without taking the Lesbos horse into account, two distinct groups are defined, calling into question the existence of allometry, the effect of size on the bony labyrinth's shape should, in this case, be explored within both groups. We report a potential allometry for the bony labyrinth but call for confirmation of this result with better sampling for future studies. Several authors previously reported strong allometric relationships of the inner ear with body mass, such as a negative ontogenetic relationship with body mass and the surrounding cranium as a result of the ossification of the bony labyrinth being completed before or shortly after birth and therefore being already fully formed while the animal continues to grow postnatally to its adult size (Osipov et al. 2013; Schutz et al. 2014; Ekdale 2016; Braga et al. 2019; Costeur et al. 2019).

In contrast to Clavel et al. (2021), who reported no bony labyrinth shape differences within the equid species based on their geometric morphometric data, our results based on traditional morphometric data show that the different shape spaces of the Aegean and Japanese horses do not overlap in any of the PCA plots, indicating unique bony labyrinth shapes for each group in our sample. Such a result suggests that there may be a phylogeographic signal at this intraspecific level for *Equus caballus*, as reported for *Homo sapiens* (Ponce de León et al. 2018), even this result should be taken with great caution because the bony labyrinth sample comprised only 3 Japanese horses. This difference in bony labyrinth shape also suggests that traditional morphometric data, despite not being directly comparable to geometric morphometric data, could also detect fine signals of variation among groups, when appropriate variables are chosen.

Variation in bony labyrinth morphology is associated with differences in the auditory (i.e., cochlea) and locomotory (i.e., canals) functions, although we must be cautious when making speculative assumptions on the reason behind certain changes, since there are numerous additional explanations to such observations (e.g., Evin et al. 2022), such as drift, skull morphology changes, different evolutionary history, and bottleneck or crossbreeding events. The analysis of the inner ear structures should be compared with breeds that have not undergone nanism in future studies, as we have done for the cranium.

### The shape of the braincase endocasts

Although we expected a high proportion of the shape variance to be explained by size, it is higher than expected. The shape spaces related to the braincase measurements only overlap in the allometry-free PCA plot (Fig. 4I), except for the Yonaguni and Misaki groups overlapping in the log shape ratios PCA (Fig. 4H). There is a segregation of the Aegean and Japanese brain shapes along PC1, when data are standardized (Fig. 4H), that disappears when removing the effects of allometry as well (Fig. 4I). Therefore, the overall shapes of the Aegean and Japanese horse brains are not significantly different in our dataset, if we consider allometric effects.

Certain brain regions of some breeds are hypothesized to have been influenced by artificial selection, in relation to specific uses of those breeds (Hanot et al. 2021)—this has yet to be tested. We observed a large difference in measurements to be the angle between the olfactory bulb and the frontal lobe, ranging from 14° in the Rhodes F specimen (NMBE 1088738) up to 72° in the Skyros IV specimen (NMBE 1088731), with the other specimens taking up intermediate angles. The height of the olfactory bulbs in relation to size is comparable among the populations, but the width of the olfactory bulbs in log shape ratios is typically lower in the Japanese breeds, except for the Tokara breed’s ratio being as large as in the Aegean ones. This angle, together with the Cerebellum–Spinal Cord Angle, seem to drive the expansion into the negative PC1 and positive PC2 quarter of the log shape ratios PCA (Figs. 4H, 5C).

Many measurements separate the Aegean and Japanese breeds in the standardized data (Figs. 4H, 5C), such as the maximal intercanal complex width. The Aegean specimens all have higher widths than the Japanese specimens. We cannot ascertain if this difference is population-specific or not, because this measurement is one that we defined, and we have no comparable data for Przewalski’s horses from Danilo et al. (2015) or any other horse population.

We cannot make inferences of any biological implications of these observation with skeletal data alone. Perhaps olfactory bulb shape and size variation reflect differences in olfactory perception, but more work in this field needs to be done. Apart from a possible effect of domestication, they could also be an artifact of the respective populations’ histories and past bottlenecks, or differences in ontogeny. Nevertheless, in our sample, we highlight extreme variation in the anatomy of the olfactory bulbs among different horse populations, as well as a significant geographical difference in intercanal complex width, demonstrating the potential of endocast morphometrics to make inferences about intraspecific and inter-breed differences for domesticates.

## Conclusions

We show that size has a significant influence on cranial, bony labyrinth, and brain shape in small island-living horses. When excluding the effects of allometry, all small island-living breeds and varieties included in this study show an overall analogous cranial shape, as they all follow the same allometric trajectory and show no significant differences between groups in the allometry-free morphospace. However, a more detailed shape analyses did reveal differences among breeds and varieties, highlighting that small size does not result in an identical shape, and leaving room for other factors to play a small role as well.

Our endocast reconstructions show variation among horse populations, especially regarding the positioning of the olfactory bulbs and the intercanal complex width, even for a small sample size and among the three Aegean (Lesbos, Skyros, Rhodes) and six Japanese (Hokkaido, Misaki, Noma, Tokara, Tsushima, Yonaguni) populations.

While this study explores the observable intraspecific variation of each method applied to a limited sample of horse crania from the Aegean and Japanese archipelagos, a comprehensive study including more samples of bony labyrinth and endocast models from different island (Fig. 1) and mainland settings could capture more of the variability of horse morphology. Such studies could facilitate our understanding of the processes of convergence in morphological divergence and help evaluate the effect of drift and the environment, which could prove relevant in the fields of bioarcheology, domestication studies, taxonomy, and evolution.

### Supplementary Information

Below is the link to the electronic supplementary material.

Supplementary file1 (XLSX 23 KB)

Supplementary file2 (TIF 67838 KB)

Supplementary file3 (DOCX 31 KB)

Supplementary file4 (XLSX 10 KB)

Supplementary file5 (XLSX 57 KB)

Supplementary file6 (TIFF 11256 KB)

Supplementary file7 (TIF 1813 KB)

Supplementary file8 (DOCX 19 KB)

## Data Availability

CT-scans and 3D Model data are available as.ply files and reduced tiff stacks on MorphoSource (https://www.morphosource.org/projects/000598941?locale=en), and Supplementary Materials are provided in association to the present article. Captions for the Supplementary Materials are available in Supplementary Material 8.
