# Supplementary material for: Insular dwarfism in horses from the Aegean Sea and the Japanese archipelago
Source: Mamm Biol. 2024 Mar 29;104(4):345–61. doi: 10.1007/s42991-024-00408-4 (PMC11281976; doi:10.1007/s42991-024-00408-4)
Supplement: Supplementary file 3 — Supplementary file3 (DOCX 31 KB) [file 42991_2024_408_MOESM3_ESM.docx]

**Supplementary Material 3**: Additional historical information on the Aegean and Japanese horses.

**The Aegean horses**

The Skyros and Rhodes horses are notoriously absent from Ancient and Medieval Greek texts. For example, they are nowhere mentioned in Aristotle’s (384-322 BC) detailed zoological texts, neither in subsequent relevant Greek nor Romans scripts. Another, younger, prominent example is the zoological work of the Byzantine emperor Constantine VII Porphyrogennetos (tenth century AD), who gathered encyclopaedical information on many subjects in his palace in Constantinople. In his zoological works, he cited much information from Aristotle, Aelianus, and subsequent writers (see Lambros 1895). Constantine discussed extensively about sizes, shapes, colours, behaviours, and various other features of horses, originating from numerous regions (not only from his empire but also from far away) but he did not mention anything about Skyros or Rhodes (Lambros 1895). At the same time, Constantine was apparently familiar with animals from Skyros as, citing Aelianus (second-third century AD), he highlighted the goat from Skyros as "producing far more milk than any other goat" ("αἱ Σκύριαι αἶγες γάλα ἀφθονώτατον παρέχουσιν ὃσον οὐκ ἂλλαι αἶγες"; Lambros 1895:134).

One of the oldest written evidence of horses on Skyros mentioned in the literature was that of a marriage contract from 1735, listing a horse as a part of the dowry (Dimitriadis, 1937). Reports of horses on the island occurred subsequently in nineteenth century literature (Heldreich 1878). All other sources appear to be too cryptic to be certainly attributed to the Skyrian breed. Even less is known about the Rhodes variety’s origin and age. There are mentions of traditional festivals including horses on Rhodes, possibly in form of a horseshow or chariotry (Arnold 1936), but other usages for the horses on the island have not been mentioned. Archaeological evidence of small horses on Rhodes exists in form of a horse buried together with humans dating back to the Bronze Age (Masseti 2012) and ancient Greek coins (e.g., Ashton 2001) as well as some plate fragments and vases depicting small horses that were found on the island, roughly dating back up to 600 BC (Villing & Mommsen 2017). It has been mentioned that the locals in Rhodes were calling their horse population as “Midili”, which would hint towards an origination of this breed from the Lesbos horse (Vezzani 1929; Kostaras 2020). The presence of small-sized individuals on Lesbos has been reported by European travellers since the middle of the 16th century (see Kritikos 1994), however, it is unclear if the few still existing feral individuals in Rhodes (Kostaras 2020) are descendants of these animals.

Nowadays, Greece is home to six local, officially registered horse breeds, defined and named by their respective supposed regions of origin: The Messara, the Penia, the Pindos, the Thessaly, the Andravida, and the Skyros Small Horse (Apostolidis et al. 2001; Hendricks 2007; Bömcke et al. 2011; Amalthia 2020). Additionally, there are several small, non-descript populations that are considered their own breeds by locals in some regions, such as the Macedonian Pacer and the Rhodes (or Rodos) Small Horse (Giantsis et al. 2018; Kostaras 2020).

Traditionally, the Skyrian Horses were captured during the time of threshing by local farmers to work on their fields, and released back into the wild, where they would remain in the mountainous terrain of the island for the rest of the year, leaving them under the influence of natural selection (Dimitriadis 1937; Hendricks 2007; Masseti 2012). With the rise of industrialisation, these traditions declined and with them the number of semi-wild horses on Skyros (Apostolidis 2001; Hendricks 2007; Kostaras 2020). The introduction and release of other grazing animals on the island, such as goats, donkeys, and mules, has become another threat to Skyros’ natural diversity. It is estimated that today only a few hundred individuals exist worldwide, in particular 180 living in Skyros and in addition, around 200 in farms in Thessaloniki, Corfu, Scotland, and France (M. Trachanas, pers. comm., February 2023). Among them, only 160 purebred individuals are known (Kostaras 2020). Nothing is less certain when it comes to question the abundance of the Rhodes horses and the Horse of Lesbos Island, the former not being officially recognized as a breed (but suggested as distinct – Amalthia, 2020), and the latter still being considered extinct until recently (Kostaras 2020).

Unlike the other native horse breeds in Greece, which reach average wither heights of around 130-155cm, (Hendricks 2007; Giantsis et al. 2018), the Skyrian horse measures only 102-115 cm (Kostaras 2020), more specifically, 109 cm for males and 107 cm for females on average (Bömcke 2011). In addition, physical descriptions of the Rhodes horses are few. Similarities of the Rhodes horse to the Sardinian “dwarf” form and the Shetland pony have been discussed (Taibell 1930). The Rhodes horse has a wither height reaching around 110 cm (Kostaras 2020). Regarding morphological variation, Dimitriadis (1937) provided morphometric measurements, a detailed physical description of the Skyrian breed, and comparisons with a variety of other breeds using simple statistics. The external descriptions of Dimitriadis (1937) match the breed’s standard description of 2013 (Brown et al. 2013): external characters have not changed substantially in the past century. More precisely, Dimitriadis (1937) noticed strong resemblances to the extinct Veglia pony of Croatia, the Exmoor pony, the Shetland pony, and to some extent also the Icelandic horses. Phenotypic resemblances of the Skyrians to the Exmoor pony and the Caspian horse, considered one of the most ancient horse breeds (Hendricks 2007; Brown et al. 2013) have been investigated (Brown et al. 2013), but close ties to either have been rejected (Brown et al. 2013). However, a previous study by Clauss et al. (2022), using some of the specimens used in our study, found similar proportions of the Skyros and Rhodes horse mandibles with other very small breeds, namely the Falabella and Shetland horses. Only a few pure-bred individuals (14, of which seven males and seven females) are known to exist as of November 2023 (I. Levendis, pers. comm.), all of them living in the farm of the Phaethon - Association for the Protection of the Small-sized Horse of Rhodes (town of Archangelos, Rhodes).

From molecular advances, genetic analyses of the Skyros horses have confirmed the breed’s uniqueness in terms of a large mean genetic distance from the other Greek breeds by investigating random amplified polymorphic DNA (RAPD) markers (Apostolidis et al. 2001) and microsatellite markers (Bömcke et al. 2011). They have shown to be different even from the relatively small Pindos Horse from the Greek mainland. Some studies on the genetics of horse gait behaviour have included samples of Rhodes horses (Promerová et al. 2014; Staiger et al. 2017), but to our knowledge, no other modern conceptualized studies on genetics have been performed on them. Consequently, we know of gene flow in the Greek breeds (Apostolidis et al. 2001) but to our knowledge, there is no study which would confirm close affiliations and a common ancestry across the Archipelago.

**The Japanese horses**

In Feudal Japan (1185-1603), horse breeders sent large-sized horses to the samurai (military nobility) and the smaller ones were sold to farmers and transporters (S. Ohdachi, pers. comm.). Consequently, both larger and smaller-sized horses were in demand and had their own purposes at the time. A major bottleneck event occurred towards the end of the 19th century: During the Meiji Era (1868-1912) the native Japanese horses were forced to be bred with larger, Western horses for military purposes. Thus, the pure lineages of traditional Japanese horses almost went extinct, except on small, remote islands and Hokkaido - a frontier region without strict laws and comparable to the "Wild West" of the USA at the time. After World War II, smaller horses were favored by residents of small remote islands due to easy handling (S. Ohdachi, pers. comm.). Until the mid-last century, horses were frequently used in farming and packing, but as they became redundant and more costly than machines, their numbers dropped significantly. This shows that most of the strong bottlenecks and selection initiated by humans on the Japanese horse breeds have occurred relatively recently, within the last century and a half.

The Hokkaido is claimed to stem from the Nanbu, an extinct breed from the Tohoku area and closely related to the Kiso (Tozaki et al. 2019) around the 13th century. Japanese settlers expanding their territory up to Hokkaido brought their horses with them in warmer months but left them to survive on their own during the harsh winter months, when the settlers returned to Honshu until spring (S. Ohdachi, pers. comm.). This made the Hokkaido breed stock naturally hardy and strong, thriving under severe conditions and even getting used to low-nutrient vegetation, such as dwarf bamboo or horsetail. The Hokkaido are also known as the Dosanko, which is also used for the people living on Hokkaido. They are still used in traditional sports, and many individuals are natural pacers. While all Japanese breeds show a relatively low heterozygosity, ranging from 0.1581 in the Tokara breed, to 0.2666 in the Hokkaido. The Hokkaido breed maintaining the highest amount coincides with the fact that they have the largest population size of all native Japanese breeds (Onogi et al. 2017; Tozaki et al. 2019).

The origin of the Kiso breed is unknown, but it likely was founded from an originally imported stock of Mongolian horses in the Kiso area of the Nagano Prefecture. It was apparently first mentioned in a sixth-century document about a region called Kiriharanomaki (now Nagano), according to a professor of Agriculture at Tokyo University called Okabe and an official at the Ministry of Agriculture by the name of Ishikazi in the 1940s (Hendricks 2007). Originally used for farm work, it became popular for military use in the late Edo Era (1600-1867), and subsequently bred with foreign breeds in the Meiji Era (1868-1912) to increase body size as a result of their inferiority to foreign breeds. One stallion, surviving the rigorous breeding program of the imperial Japanese army, sired a son in 1951, which is said to be the founding sire of the modern Kiso (Hartley Edwards 2016).

The Misaki breed, that has been rarely controlled until this day, was founded around 1600 on Kyushu, bred on a semi-feral state (Tozaki et al. 2019). Only once, in the early Taisho Era, a western trotter stallion of unknown breed was introduced into the population but had apparently no large influence on the Misaki (Hendricks 2007, Tozaki et al. 2019). During the Edo period, they were mainly used in war and agriculture, while retaining the characteristics of the old Japanese breeds (Hendricks 2007).

The Noma breed is supposed to have arisen from an unknown founding population in 1635 on Shikoku (Tozaki et al. 2019). Entrusted to the local farmers by the feudal lords of Matsuyama, the Hisamatsu family, they were used for the transport of agricultural items in steep-sloped regions, for which they have been selected for small size, especially in more recent times (S. Ohdachi, pers. comm.). Despite the breed improvement policies of the government, efforts were made to keep them pure for preservation (Hendricks 2007).

In 1897, as people moved to Takara Island– one of the Tokara Islands – from Kikai Island, they brought along 10 horses that made up the founding population of the Tokara breed. This breed was used for farm work, transportation and sugar cane processing (Hartley Edwards 2016). In 1943, their population peaked at 100 animals, which declined in the 1960s again due to mechanization. During this time, some of them were moved to the main island to Kagoshima, where some of them remain (Hendricks 2007). It is not known where the Kikai population originated from, but genetic analyses suggest a common ancestry for the Tokara, Miyako and Yonaguni breeds (Tozaki et al. 2019).

The island of Miyako was known as a horse breeding area for centuries, and horses are said to always have roamed the island. Not much is known about their origins, other than their genetic closeness to the Tokara and Yonaguni (Tozaki et al. 2019). In order to make them stronger for farm work, they were crossed with larger, foreign stallions during and after World War II (Hendricks 2007). Although their peak population size in 1955 was over 10’000 animals, nowadays only a handful remain. They are also the only native Japanese breed not represented in our study.

Although claims of Yonaguni being populated during the Jōmon period over 2’000 years ago have been made, genetic analyses have confirmed them to be closest related to the geographically close Miyako and Tokara breeds (Tozaki et al. 2019). However, a journal about Yonaguni Island, written by Koreans in 1479, mentioned islanders keeping both cattle and horses (Hendricks 2007), which begs the question if the population in 1479 was the same as the modern Yonaguni, or if the island had a second introduction of horses at a later point in time. In any case, this would be the oldest record of horses on any of the Ryūkyū Islands. Despite the Yonaguni being spared from the improvement breeding programs in the past, some of them sporadically show white markings, which might be an indication that crossbreeding could have occurred in the past.

REFERENCES

Amalthia (2020) Greek Domestic Breeds - A hidden treasure. Amalthia - Network for the Protection of Greek Indigenous Farm Animals, Athens

Apostolidis AP, Mamuris Z, Karkavelia E, Alifakiotis T (2001) Comparison of Greek breeds of horses using RAPD markers. J Anim Breed Genet 118:47-46. https://doi.org/10.1111/j.1439-0388.2001.00272.x

Arnold IR (1936) Festivals of Rhodes. Am J Archeol 40(4):432-436. https://doi.org/10.2307/498795

Ashton RHJ (2001) The coinage of Rhodes. 408-c. 190 B.C. In: Meadows, A. and K. Shipton (eds.), Money and its Uses in the Ancient Greek World. Oxford University Press, Oxford, pp 79-116

Bömcke E, Gengler N, Cothran EG (2011) Genetic variability in the Skyros pony and its relationship with other Greek and foreign horse breeds. Genet Mol Biol 34(1):68-76. https://doi.org/10.1590/S1415-47572010005000113

Brown SA, Moore-Colyer MJS, Hannant D (2013) Phenotypic analyses support investigations of phylogeny in the Skyrian pony and other breeds. Biosci Horiz 6:hzt010. https://doi.org/10.1093/biohorizons/hzt010

Dimitriadis JN (1937) Das Skyrospony. Ein Beitrag zum Studium der Pferde Griechenlands. Zeitschrift für Züchtung. Reihe B, Tierzüchtung und Züchtungsbiologie einschliesslich Tierernährung 37(3):343-385. https://doi.org/10.1111/j.1439-0388.1937.tb00077.x

Giantsis IA, Diakakis NE, Avdi M (2018) Genetic Composition and Evaluation of the Status of a Non-descript Indigenous Horse Population From Greece, the Macedonian Pacer. J Equine Vet Sci 71:64-70. https://doi.org/10.1016/j.jevs.2018.10.003

Hartley Edwards E (2016) The Horse Encyclopedia. Dorling Kindersley Limited, London

Heldreich T (1878) La faune de la Grèce. Athens

Hendricks BL (2007) International encyclopedia of horse breeds. University of Oklahoma Press, Norman, Oklahoma

Kostaras N (2020) Greek Equine Breeds. In: Greek Domestic Breeds - A hidden treasure. Amalthia - Network for the Protection of Greek Indigenous Farm Animals, Athens, pp 37-67

Kritikos N (1994) [Anatomical and physiological parameters of the small-sized horse breed from Skyros]. BSc Thesis, Agricultural University of Athens

Lambros SP (1895) Excerptorum Constantini de Natura Animalium Libri Duo – Aristophanis Historiae Animalium Epitome, Subiunctis Aeliani Timothei Aliorumque Eclogis. Typis et Impensis Georgii Reimer, Berlin, 282 pp.

Masseti M (2012) Atlas of terrestrial mammals of the Ionian and Aegean islands. De Gruyter, Boston & Berlin, pp 207-220. https://doi.org/10.1515/9783110254587

Onogi A, Shirai K, Amano T (2017) Investigation of genetic diversity and inbreeding in a Japanese native horse breed for suggestions on its conservation. Anim Sci J 88:1902–1910. https://doi.org/10.1111/asj.12867

Promerová M, Andersson LS, Juras R, Penedo MCT, Reissmann M, Tozaki T, Bellone R, Dunner S, Hořín P, Imsland F, Imsland P, Mikko S, Modrý D, Roed KH, Schwochow D, Vega-Pla JL, Mehrabani-Yeganeh H, Yousefi-Mashouf N, Cothran EG, Lindgren G, Andersson L (2014). Worldwide frequency distribution of the ‘Gait keeper’ mutation in the DMRT3 gene. Anim Genet 45:274–82. https://doi.org/10.1111/age.12120

Staiger EA, Almén MS, Promerová M, Brooks S, Cothran EG, Imsland F, Jäderkvist Fegraeus K, Lindgren G, Mehrabani Yeganeh H, Mikko S, Vega-Pla JL, Tozaki T, Rubin CJ, Andersson L (2017) The evolutionary history of the DMRT3 ‘Gait keeper’ haplotype. Anim Genet 48:551–559. https://doi.org/10.1111/age.12580

Taibell A (1930) Sul cavallo di Rodi. It J Zool 1(1):127-130. https://doi.org/10.1080/11250003009434819

Tozaki T, Kikuchi M, Kakoi H, Hirota K, Nagata S, Yamashita D, Ohnuma T, Takasu M, Kobayashi I, Hobo S, Manglai D, Petersen JL (2019) Genetic diversity and relationships among native Japanese horse breeds, the Japanese Thoroughbred and horses outside of Japan using genome-wide SNP data. Anim Gen 50:449-459. https://doi.org/10.1111/age.12819

Vezzani V (1929) Rodi e il suo problema zootecnico. Istituto zootecnico e caseario per il Piemonte Torino, rivista “Natura”, Milano

Villing A, Mommsen H (2017) Rhodes and Kos: East Dorian Pottery Production of the Archaic Period. The Annual of the British School at Athens 112:99-154. https://doi.org/10.1017/S0068245417000053
