# Supplementary material for: Insular dwarfism in horses from the Aegean Sea and the Japanese archipelago
Source: Mamm Biol. 2024 Mar 29;104(4):345–61. doi: 10.1007/s42991-024-00408-4 (PMC11281976; doi:10.1007/s42991-024-00408-4)
Supplement: Supplementary file 8 — Supplementary file8 (DOCX 19 KB) [file 42991_2024_408_MOESM8_ESM.docx]

**Electronic Supplementary Materials**

**S1** Excel file of small horse breeds of the world (< 146 cm average withers height), based on information from Hendricks (2007) and Hartley Edwards (2016). Abbreviation: hh, hands high (= old measuring unit for equine height).

**S2** Map indicating countries in which small horse breeds occur, based on S1. Map created on mapchart.net

**S3** Additional historical information on the Aegean and Japanese horses.

**S4** Excel file of all Aegean horse material collected. Abbreviation: NMBE, Natural History Museum Bern.

**S5** Full spreadsheet with measurement data in millimeters. Cranial measurement data complemented by distance measurements from Dimitriadis (1937) and by distances calculated from landmark data published in Heck et al. (2018). Measurement descriptions according to Table 2. Abbreviations (modified from Heck et al. (2018): ID_String (unique identifier of each specimen), Museum (A = Argentina, B = Berlin, BOT = Botanical Garden of Hokkaido University, EM = Equine Museum of Japan, H = Halle, JND = JN Dimitriadis, K = Kiel, KU = Kagoshima University, NMBE = Natural History Museum Bern, NU = Nihon University Fujisawa Campus, OU = Obihiro University, SO = Satoshi D. Ohdachi Personal Collection, TU = Tsukuba University, V = Vienna), ID (identifier used at the museum or given by us), Group (Ch = Cheju Pony, E = Exmoor Pony, Fa = Falabella, H = Horse, Ho = Hokkaido, I = Icelandic, K = Kiso, L = Lesbos, M = (Japanese) Midget, Mi = Misaki, No = Noma, P = Przewalski’s Horse, Po = Undefined Pony (from Japan), R = Rhodes, S = Shetland, Sc = Scottish Pony, Sk = Skyros, To = Tokara, Ts = Tsushima Horse, Yo = Yonaguni, Yu = Yuigahama-minami Horse), Breed (aaa = not a domesticated horse, ahb = Ancient Breed (Roman period), ano = Anglo-Norman, arb = Arab, bif = Birkenfelder, blg = Belgian Draft, bos = Bosnian Pony, cds = Clydesdale, che = Cheju Pony, exm = Exmoor Pony, fab = Falabella, gbh = Galician Farm Horse, grb = Grisons (Graubündner), grp = German Riding Pony, han = Hannoverian, hny = Hackney, hok = Hokkaido, hol = Holstein, hun = Hungarian, huz = Huzule, ice = Icelandic, ind = Indian Pony, kdr = Kladrubian, kis = Kiso, kon = Konik, kos = Kosarian, les = Lesbos, lpz = Lipizzan, mid = (Japanese) Midget, mis = Misaki, mon = Mongolian, nom = Noma, nor = Norik, odb = Oldenburgian, piz = Pinzgau, pll = Polish Farm Horse, pony = Undefined Pony (from Japan), scp = Scottish Pony, ses = Seneca Sarajevo, she = Shetland Pony, shi = Shire, sky = Skyros, stm = Styrian, suf = Suffolk, tbh = English Thoroughbred, tog = Togo Pony, tok = Tokara, trk = Trakehner, tsu = Tsushima Horse, wel = Welsh, yon = Yonaguni, yui = Yuigahama-minami Horse), Measurer (JND = JN Dimitriadis (Dimitriadis 1937), KMM = Keesha M. Ming, LH = Laura Heck (Heck et al. 2018)).

**S6** Principal component analyses of cranial shape in the investigated horses with a more detailed grouping. Cranial morphospace with raw (= form) (**A**), log shape ratios (**B**) and allometry-free shape (**C**) data. Single datapoints have been highlighted for visibility. Refer to Fig. 4A-C for further details.

**S7** Multivariate regressions of the PC1 scores from the log shape ratios PCA with log(geometric mean) for the cranial shape in the investigated horses with a more detailed grouping. Gray spectrum indicates a 95% confidence interval. Refer to Fig. 5A for further details.
